# Combating poor-quality anti-malarial medicines: a call to action

**DOI:** 10.1186/s12936-016-1357-8

**Published:** 2016-06-01

**Authors:** Quique Bassat, Marcel Tanner, Philippe J. Guerin, Kirstin Stricker, Kamal Hamed

**Affiliations:** Centro de Investigação Em Saúde Da Manhiça (CISM), Maputo, Mozambique; ISGlobal, Barcelona Centre for International Health Research (CRESIB), Hospital Clínic-Universitat de Barcelona, Barcelona, Spain; Swiss Tropical and Public Health Institute, Basel, Switzerland; University of Basel, Basel, Switzerland; WorldWide Antimalarial Resistance Network, Oxford, UK; Centre for Tropical Medicine and Global Health, Nuffield Department of Medicine, Oxford University, Oxford, UK; Novartis Pharma AG, Basel, Switzerland; Novartis Pharmaceuticals Corporation, East Hanover, NJ USA

**Keywords:** Anti-malarials, Counterfeit drugs, Falsified, Malaria, Quality control, Substandard

## Abstract

The circulation of poor-quality medicines continues to undermine the fight against many life-threatening diseases. Anti-malarial medicines appear to have been particularly compromised and present a major public health threat in malaria-endemic countries, negatively affecting individuals and their communities. Concerted collaborative efforts are required from global, regional and national organizations, involving the public and private sectors, to address the problem. While many initiatives are underway, a number of unmet needs deserve urgent and increased multisector attention. At the global level, there is a need for an international public health legal framework or treaty on poor-quality medicines, with statutes suitable for integration into national laws. In addition, increased international efforts are required to strengthen the governance of global supply chains and enhance cooperation between national medicine regulation authorities and law enforcement bodies. Increased investment is needed in innovative technologies that will enable healthcare teams to detect poor-quality medicines at all levels of the supply chain. At the regional level, a number of initiatives would be beneficial—key areas are standardization, simplification, and reciprocal recognition of registration processes and development of quality control capacity in regional centres of excellence that are better aligned with public health needs; improved surveillance methods and creation of a framework for compulsory and transparent reporting of poor-quality medicines; additional support for national medicine regulation authorities and other national partner authorities; and an increase in support for regional laboratories to boost their capabilities in detecting poor-quality medicines. It is vital that all stakeholders involved in efforts against poor-quality anti-malarial medicines extend and strengthen their actions in these critical areas and thus effectively support global health development and malaria elimination programmes.

## Background

In 2015, an estimated 214 million malaria cases occurred globally, causing approximately 440,000 deaths [1]. Nearly half the world’s population lives in areas where malaria transmission is a threat [2]. Although global malaria incidence declined by 37 % between 2000 and 2015, major obstacles remain [1]. Some of these challenges are anti-malarial and insecticide resistance, lack of sustainable funding and poor-quality medicines, which could prevent further progress or compromise gains in global malaria control.

The circulation of poor-quality medicines, namely falsified (i.e., intentional fraudulent manufacturing) or substandard (i.e., unintentional errors in manufacturing or degradation because of poor storage/handling) products is a significant barrier to the treatment of many conditions and diseases [3, 4]. Anti-infectives, particularly anti-malarials, are vulnerable targets and among the most common classes of drugs associated with quality concerns [5–10]. Such medicines pose a major public health threat in all endemic countries by simultaneously impacting at multiple levels within a population, affecting individuals and communities. At the individual level, they can cause treatment failures with prolonged or more severe sickness and death. In 2013 alone, the consumption of poor-quality anti-malarial medicines was estimated to have caused over 120,000 deaths among children younger than 5 years in 39 countries in sub-Saharan Africa [11]. There can also be financial implications for vulnerable patients and families, including loss of income and wastage of out-of-pocket expenses. At the public health level, by delivering sub-therapeutic drug concentrations, poor-quality medicines may drive the selection of drug resistance [7, 12, 13]. Furthermore, studies in malaria have shown that patients treated with poor-quality anti-malarials have a higher prevalence of gametocytes (sexual-stage parasites that can transmit from the human host back to the mosquito) post-treatment and, therefore, there is a higher chance of transmission by mosquitoes of drug-resistant parasites to susceptible populations [14]. Ineffective treatment translates as depletion of healthcare resources and could result in a loss of public confidence in drugs, pharmacies, and healthcare providers [9]. It also threatens confidence in anti-malarial programmes at a national and even global level, jeopardizing progress and future investment in malaria control and elimination [15]. Use of poor-quality medicines in clinical trials is a further important concern, as this may compromise the reputation of and confidence in effective drugs, healthcare providers, and delivery systems and misinform public policy [16].

In 2011, the World Health Organization (WHO) published evidence of a substantial problem in the quality of anti-malarial products in sub-Saharan Africa [17]. Further reports from Africa and Southeast Asia have led to international concern and prompted action [3, 5, 7, 8, 15, 18]. This paper discusses the current evidence base of the extent of the problem of poor-quality anti-malarial medicines, the underlying factors, and the feasible actions to address this key problem.

## Defining and quantifying the problem

Coordinated international actions to address the problem of poor-quality anti-malarials are hindered by (1) the paucity of accessible and reliable information on the prevalence of such medicines in circulation, and (2) the difficulty this creates in determining their real impact on public health. The lack of internationally accepted definitions makes the evaluation of the impact of their circulation even more complex.

### Definitions

National and international authorities use an array of terms relating to medicine quality, and differences exist in the definitions used [7]. The WHO has used the term ‘substandard, spurious, falsely labelled, falsified and counterfeit’ (SSFFC) to encompass the range of poor-quality medicines [19]. However, it is important to differentiate between the different forms and levels. In recent years, attention has focused on the key distinction between ‘falsified’ and ‘substandard’ medicines [3, 7, 18]. A ‘falsified’ medicine is deliberately and fraudulently mislabelled with regard to its identity or source (i.e., with criminal intent) [20]. Falsification can apply to both branded and generic products and refer to medicines in which the active pharmaceutical ingredients (API) could theoretically be correct, wrong, insufficient, or absent, or be a result of fake packaging. This definition of falsification can also apply to the term ‘counterfeit’. However, this term has been primarily associated with intellectual property and trademark protection, rather than public health and health outcomes, the prime consideration when defining the quality of medicines [5]. A ‘substandard’ medicine is a genuine drug product that does not meet quality specifications because of manufacturing error or that degrades over time within the recommended shelf-life [18, 20]. The specifications typically include a defined content of the API and/or the formulation.

### Prevalence of poor-quality anti-malarial medicines

Estimates of the prevalence of poor-quality medicines vary according to the sampling and analytical methods used, and reliable, comparable data are sparse [8, 15]. In 2008, the WHO coordinated a survey of the quality of artemisinin-based combination therapy (ACT) and sulfadoxine/pyrimethamine medicines in six sub-Saharan African countries that had received WHO support to strengthen regulatory controls [17]. Overall, 76 of 267 samples (28.5 %) failed to comply with WHO specifications. Extreme deviations from the specifications, likely to be associated with negative health outcomes, were found in 11.6 % of samples. In 2009, a parallel study of 197 samples was conducted collaboratively by the WHO and the United States Pharmacopeia Drug Quality and Information Program, in which 44 % of samples from Senegal failed quality control tests, along with 30 % from Madagascar and 26 % from Uganda [21].

The WorldWide Anti-malarial Resistance Network (WWARN) Anti-malarial Quality Literature Surveyor [22], an open-access database of published surveys and reports, aims to contribute to the understanding of the evidence base and clarify how existing data can inform relevant public health policies [8]. According to a systematic review of 251 studies in the database (1946–2013), 2813 of 9348 (30.1 %) sampled anti-malarial products failed chemical or packaging quality tests: 1107 (39.3 %) were classified as falsified, 66 (2.3 %) as substandard, and 1640 (58.3 %) as unspecified poor-quality (i.e., without evidence for further categorization) [8]. No publicly available reports on the quality of anti-malarial medicines were found for 63 of the 104 (60.6 %) malaria-endemic countries, and important weaknesses and inconsistencies were also evident in study methods and reporting.

Several recently published large surveys [23–25] and reports on drug seizures in Africa and Southeast Asia [16, 26] warrant continued concern with regard to the prevalence of substandard medicines that fail tests of API content (Table 1). Some surveys conducted between February 2010 and February 2013 have also found artesunate monotherapy tablets in circulation in sub-Saharan Africa [23, 27, 28] and Southeast Asia [26]. This may be partly due to the difference in expense, as the total cost of ACT is approximately double that of monotherapy. Additionally, analysis suggests that monotherapy is used mainly in self-treatment by adults purchasing from patent medicine vendors, which indicates the need to continue and extend public awareness of ACT [29]. Recent reports suggest such campaigns have been relatively successful in Cambodia [30] and Lao People’s Democratic Republic (Laos) [31] as the use of artemisinin monotherapy has become less common following its global ban in 2006 [32]. Political awareness has also increased and specific actions have been carried out related to artemisinin resistance emergence in this region.

**Table 1:** Summary of selected recently published surveys of anti-malarial medicines quality in Africa and Southeast Asia

Region/country and year	Product and number of samples	Source(s) and sampling	Key results
Africa			
Tanzania 2010 [24]	Artemisinin-containing N = 1737	Source: private retail outlets Sampling: nationally representative sample	All samples contained an API Artemisinin-containing derivative: 4.1 % substandard (outside the 85–115 % API range) PQ drugs had 10 % of the odds of being poor-quality vs non-PQ Partner drugs 12.1 % substandard PQ had 4 % of the odds of being poor-quality
Ghana, Togo 2010–2011 [28]	Artemisinin-containing N = 132	Source: retail outlet Sampling: convenience sample	Only one sample lacked an API Combination products: 83.7 % (outside 90–110 % API) Monotherapy: 57.9 % substandard
Nigeria 2012–2013 [23]	Artemisinin-containing N = 3024	Sources: pharmacies (35.6 %), patent medicine vendors (60.6 %), public health facilities (3.6 %), market stalls (0.2 %) Sampling: mostly mystery client (63.5 %), then overt (6.6 %) or convenience (29.9 %)	9.2 % poor-quality: 6.8 % substandard; 1.3 % degraded; 1.2 % falsified Convenience sampling yielded a significantly higher prevalence of poor-quality
Democratic Republic of Congo 2014 [25]	Artemisinin-containing N = 238^a^	Source: private licenced wholesalers Sampling: cross-sectional, mystery client	21 % were found non-conform for the content in API 48 % were under-dosed in artemether
Southeast Asia			
Cambodia 2010–2011 [30]	Artemisinin derivatives N = 291	Sources: private health provider Sampling: mystery client	All samples contained an API Overall: 31.3 % substandard (outside range of 85 % and < 115 %) 24.7 % were expired Artesunate tablets: 25.8 % (60/233) substandard Co-blistered mefloquine: 73.4 % (149/203) substandard Considering both drugs: 77.3 % (157/203) substandard
Lao People’s Democratic Republic (Laos) 2012 [31]	Various anti-malarial medicines N = 146	Source: Private retail outlets Sampling: Cross-sectional random sample, mystery client	All samples contained an API 25.4 % substandard (outside 90–110 % API)

^a^Samples included amoxicillin, artemether/lumefantrine powder for suspension in paediatric dosage and paracetamol tablets 500 mg

## Multi-level factors and consequences

**Fig. 1:**
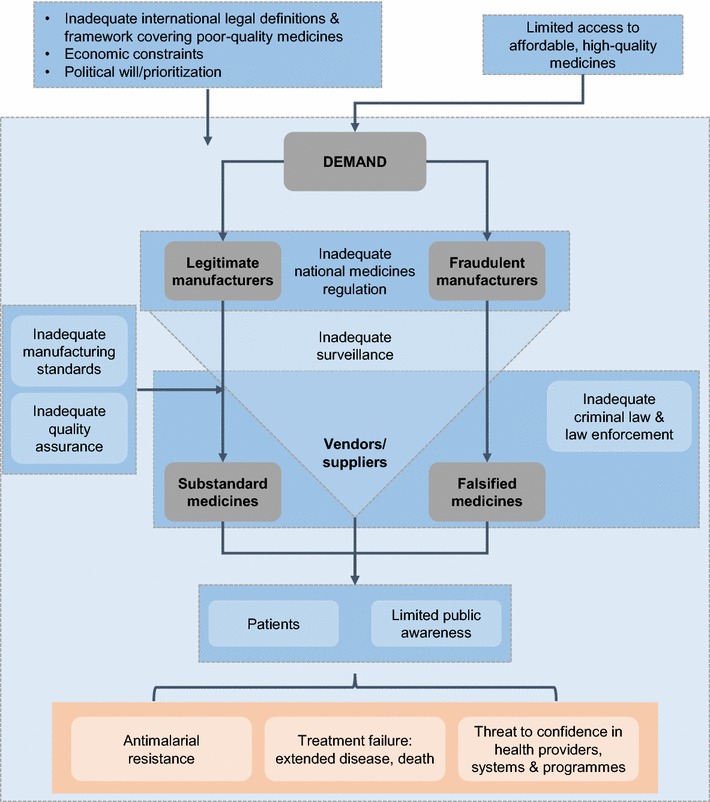
Key factors in the manufacture and circulation of poor-quality antimalarial medicines: targets for action

The circumstances that enable the manufacture and circulation of poor-quality medicines are multifactorial and are often inter-linked, impacting at the global, national, population, and individual levels (Fig. 1).

### Global

- *Lack of effective surveillance* the absence of a global system for the mandatory reporting, assessment, and dissemination of information on falsified and substandard medicines is a major obstacle to measuring the scale of the problem, raising sufficient awareness, and improving global medicines supply [33]. This problem is amplified by deficits in national-level quality surveillance in many malaria-endemic countries [34].
- *International supply chain regulation* the globalized marketplace means that medical products can be manufactured in one country, packaged in another, and supplied to others with limited international oversight of manufacturing, testing, or storage practices for legitimate medicines or the introduction of deliberately falsified products into the supply chain [7].
- *Legal* insufficient legal action against falsified and substandard (if negligence could have been corrected) medicines are facilitated by inadequate and/or unharmonized international and national laws and law enforcement against this form of fraud. The regulation of legitimate medicines manufacture is also hampered by the lack of a consistent legal framework [35].

### National

- *National regulation* the weakness of national medicine regulation authorities (NMRAs) in many countries is a central concern with regard to both falsified and substandard medicines. Documented shortfalls in national regulation in developing countries include: low prioritization of regulation within health systems; fragmentary, out-dated, and poorly coordinated regulatory frameworks; uneven and weak implementation of functions; inadequate adaptation and use of guidelines; inappropriate organizational structures (not able to ensure transparency, independence, and accountability); weak inter-sectoral collaboration nationally and among countries; shortages of qualified staff; and inadequate and unsustainable funding [34, 36].
- *Access to good-quality medicines* limitations on access to affordable good-quality treatment can drive demand for poor-quality medicines, or for monotherapy. A shortage of medicines can also lead to a reliance on unknown suppliers and illicit/uncontrolled supply chains, where the risk of poor-quality medicines may be greater. The introduction of falsified meningococcal vaccines during the 2015 outbreak in Niger illustrates the rapid capability of this organized crime [37].
- *Costs of testing for medicine quality* standard methods of testing drug reliability, such as high-performance liquid chromatography and spectroscopy, require high-cost instruments, expensive maintenance, and trained proficient staff. These constraints, in addition to less-than-optimal operational conditions, inhibit the use of these techniques in low-income malaria-endemic countries [38]. Development of portable rapid-testing methods, which are discussed in more detail in the surveillance and quality testing section below, offer the prospect of introducing less expensive and more sensitive technologies. However, the absence of a single affordable, reliable, validated, and portable technology currently remains a barrier [39].

### Population and individual

- *Anti-malarial resistance* sub-therapeutic drug concentrations may facilitate the selection and spread of anti-malarial drug resistance [7, 12, 13, 40].
- *Public confidence* ineffective treatments can result in a loss of public confidence in drugs, pharmacies, and healthcare providers, as well as in anti-malarial programmes [15].
- *Public awareness* evidence suggests that levels of public awareness about poor-quality medicines in malaria-endemic areas are sub-optimal [41]. This is particularly important given the frequency of self-medication.
- *Treatment failure* inadequate therapy leads to extended, more severe illness, and increased fatalities compared with recommended therapy [11].
- *Financial* loss of income and increased expenses for patients and families.

The success of malaria treatment has not gone unnoticed by criminal elements that desire to profit from this accomplishment by manufacturing falsified anti-malarials. Multisector collaboration involving bodies at the international and national levels is needed to address this and other factors that drive and facilitate the manufacture and dissemination of falsified and substandard medicines. These bodies include the WHO, non-governmental organizations, the scientific community, national governments, NMRAs, law-makers and law enforcement authorities (including police, customs, and the International Criminal Police Organization [INTERPOL]), public health bodies, healthcare providers, civil society, and pharmaceutical manufacturers. The following sections discuss actions underway to improve quality standards.

## Improving quality standards

### Surveillance and quality testing

Surveillance is fundamental to the planning and implementation of national malaria programmes [42]. Enhanced pharmacovigilance is needed to monitor the safety and efficacy of anti-malarial medicines and manage anti-malarial drug resistance, to ensure that the most appropriate combinations are used [42]. Currently, the quality and comprehensiveness of surveillance data on medicines quality are limited, mainly because of: the lack of a consensus in malaria-endemic countries on regulations for surveillance definitions and methods; the voluntary status of reporting requirements; and inadequate training, equipment, and funding at the level of NMRAs.

At present, specific efforts to improve surveillance are focused on survey approaches, methods linked to information sharing systems, and new analytic technologies. The WHO is developing new policies on sampling procedures and reporting medicine quality surveys. Draft recommendations [43] and guidelines [44] are in preparation to help improve monitoring and post-market surveillance. The WHO Medical Product Alert system is an important vehicle for NMRAs and other bodies to share information regarding incidents involving poor-quality medicines [45]. This reporting system started in 2013 and has received approximately 1000 reports since its launch. However, as the alerts issued by The WHO Medical Product Alert system are voluntary, sporadic, and not always public, it has been proposed that these alerts are made mandatory and included in international health regulations [33]. Timely reporting would facilitate corrective actions appropriate to the event described. The WWARN Antimalarial Quality Literature Surveyor allows users to track and evaluate the evolving evidence base of anti-malarial medicine quality via customizable maps and tables [22]. Extending the classes of medicines included in surveillance programmes may broaden the interest of potential sites.

Pharmaceutical companies also have a role in reporting any suspected poor-quality medicine, with internal policies covering verification processes and timely engagement with relevant national and global stakeholders. The 2016 Access to Medicine Index emphasizes the need for all pharmaceutical companies to have such policies [46]. Mackey et al. described 1510 Pharmaceutical Security Institute Counterfeit Incident System reports from 2009 to 2011 involving global legitimate medicine supply chain penetration [10]. This information was collected by the Pharmaceutical Security Institute, a not-for-profit membership organization of 33 pharmaceutical manufacturers [47].

The development of novel analytic technologies offers promise in facilitating surveillance and identification of suspect drugs at point-of-care by drug inspectors and law enforcement officials. Examples include Raman spectroscopy [38, 48, 49], the US Food and Drug Administration-supported counterfeit detection device (CD-3) [31, 38, 50, 51], the counterfeit drug indicator (CoDI) [38], new colorimetric assays [38], chemical colour test cards [52], and track-and-trace packaging design [53]. However, these are not yet widely affordable nor readily scalable, and further work is required to validate their use and performance in tropical contexts for anti-malarial medicines and to define their role in practice at different levels of the supply chain [3]. Ultimately, such tools can only be effective if placed in the hands of adequately resourced and trained public health staff who could report to NMRAs.

Furthermore, quality failure rates can vary owing to a lack of harmonization or agreed-upon detection standards (i.e., standardization), and criminal producers of falsified medications may adapt their formulations in an attempt to fool less-rigorous tests [54]. Therefore, suspect products may require full analysis at NMRA-certified laboratories for comprehensive investigation.

### The WHO prequalification of medicines programme

The WHO prequalification of medicines programme aims to increase the availability of quality-assured priority medicines through evaluation and inspection activities, and by building national capacity for high-quality manufacturing and monitoring [55]. Currently, this is the only global medicines quality assurance programme and, in addition to medicinal products, it also prequalifies APIs and quality control laboratories. The prequalification programme has many benefits to pharmaceutical manufacturers, including participation in international tenders, facilitated registration in some recipient countries, capacity building, technical assistance, and the benefits conferred by credibility among procurement and NMRAs.

This programme is conducted in cooperation with NMRAs. Onsite inspections are made to ensure the manufacturing site and any associated contract research organizations are compliant with WHO standards. Data from a 2008 survey support the effectiveness of the programme. Only 3 of 83 (3.6 %) tested prequalified medicines were found to be non-compliant with standards, compared with 29 of 48 (60.4 %) non-prequalified products [6]. Prequalified artemisinin-containing medicines were recently found to have 10 % of the odds of being poor-quality compared with non-prequalified medicines (Table 1) [24]. Lack of sustainable funding is a continuous challenge for this effective programme [56]. The WHO prequalification is a lengthy process and limited resources have impaired its ability to support a larger number of manufacturers; extend the range of medicines beyond that of HIV/AIDS, malaria, tuberculosis, and reproductive health; or be responsive to prequalification requests in a timely manner. Currently, the prequalification process takes a minimum of 3 months from application to approval (if the product meets all the required standards) [57].

**Fig. 2:**
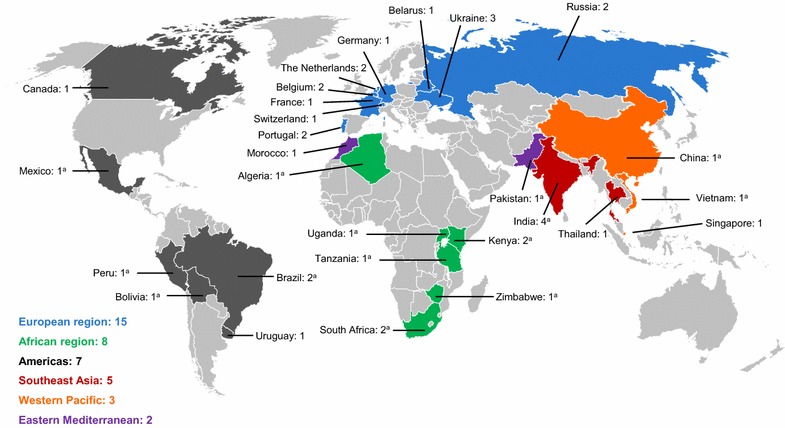
Map showing the locations of prequalified quality control laboratories [56]. ^a^Countries with endemic malaria (ongoing) [1]

A corresponding process is used to prequalify quality control laboratories [17], although responsibility for formal approval of laboratories rests with NMRAs. The WHO invites NMRAs to observe on-site inspections and notifies NMRAs when laboratories achieve prequalification. The main limitation of this initiative is the small number of prequalified laboratories: currently there are 40, of which 21 are in malaria-endemic countries and only seven exist in five countries in sub-Saharan Africa (Fig. 2) [58]. Localization of these qualification processes should be the long-term goal, and funding should be made available so that the precertification and qualification processes can occur within each country.

### Other initiatives

There are many initiatives that aim to support the global use of quality medicines, and comprehensively detailing each of these would be beyond the scope of this paper. However, some key examples are described.

The WWARN quality assurance/quality control programme has been initiated and has assumed a leadership position in helping local laboratories to assess and improve their assay quality [59–61]. This programme has two major components: a proficiency testing programme and a reference material programme.

The ACT consortium has four key research themes, one of which is quality. It states that the impact of improved delivery of ACT will be undermined if the drugs are of suspect quality owing to counterfeiting, substandard manufacturing, or degradation from poor or prolonged storage [62].

The ‘Promoting the Quality of Medicines in Developing Countries’ (PQM) programme is a United States Pharmacopeial Convention (USP) and United States Agency for International Development (USAID) partnership that helps developing countries address critical issues related to poor-quality medicines [63]. Through an array of activities, including medicine quality monitoring, education campaigns, and assistance in legislation and regulations [64], the PQM programme strives to accomplish four key objectives: to strengthen quality assurance and quality control systems; increase the supply of quality-assured medicines; combat the availability of substandard and counterfeit medicines; and provide technical leadership and global advocacy [63].

There are several other programmes launched by the USP, including the Regulatory Standards Assistance Program, which provides developing countries with tools to increase their capacity to test the quality of medicines for their citizens. In 2011, the Regulatory Standards Assistance Program began with five countries in Africa; it has since expanded to include more than 35 countries in Africa, Asia, and Latin America and the Caribbean. This programme provides participating countries with a package of reference standards selected by the participating country from the USP’s catalogue, documentary standards, and analytical data to test medicines to strengthen the reliability of quality control tests.

The Global Fund is a financing institution that supports programmes run by local experts against HIV/AIDS, tuberculosis, and malaria. Global Fund grants may only be used to procure pharmaceutical products in accordance with the standards prescribed in the Global Fund quality assurance policy. One of these standards requires anti-malarials to be prequalified by the WHO Prequalification Programme or authorized for use by a Stringent Drug Regulatory Authority [65, 66].

## Health policy issues

### National registration process

Effective regulation to ensure the quality of anti-malarial medicines being manufactured, imported, and supplied within a country is fundamental to prevent the circulation of falsified and substandard medicines. As part of its Global Malaria Strategy, the WHO recently urged all NMRAs in endemic regions to remove all inappropriate and ineffective anti-malarial medicines from healthcare facilities, pharmacies, informal providers, and private sector markets. NMRAs are also urged to regulate against the production, marketing authorization, export, import, and use of oral artemisinin-based monotherapies [42].

The WHO has specifically highlighted the need to rapidly build greater regulatory capacity in African countries, in terms of management structures, technical expertise, inter-country harmonization, and collaboration, as well as physical resources [34, 36]. The obstacles to progress in regulatory control have been well summarized and a series of actions have been proposed (Table 2) [36]. The regional strategy on regulation of medical products in the African region will be reviewed at the 66th session of the WHO Regional Committee for Africa (Addis Ababa, Federal Republic of Ethiopia, 27–30 August 2016). The WHO Prequalification of Medicines Programme supports NMRAs to improve manufacturing standards for legitimate medicines, providing training and tools, as well as via the prequalification certification itself. However, considerable expansion of the laboratory prequalification programme is clearly needed in malaria-endemic areas (Fig. 2). The US Institute of Medicine recommended that international funding should be made available to assist pharmaceutical manufacturers who wish to upgrade to international standards [7].

**Table 2:** Actions proposed by the WHO Regional Committee for Africa [36]

Prioritize the development of medical products regulation
Strengthen the coherence and performance of the medicines regulatory system (including dialogue among stakeholders)^a^
Adapt and use guidelines in line with WHO recommendations
Increase implementation of regulatory functions
Enhance the status of NMRAs
Institute sustainable mechanisms to effectively manage conflicts of interest
Strengthen inter-sectoral collaboration between relevant stakeholders
Ensure availability of qualified human resources for regulation of medical products
Ensure adequate and sustainable financing of the medicines regulatory system
Improve collaboration, coordination, and harmonization of medical products regulation

^a^Defined as NMRAs, manufacturers, traders, consumers and other representatives of civil society, health professionals, researchers, police, customs, the judiciary, governments, and parliamentarians

### National Medicines Regulatory Authorities (NMRAs) and World Health Organization (WHO)

Weak international collaboration and harmonization between NMRAs (e.g., for mutual recognition of marketing authorizations) has been identified by WHO as a problem, especially in Africa (Table 3) [36]. Surveyed pharmaceutical companies have indicated that varying national regulatory requirements are a barrier to registering and supplying medicines to African countries [67]. More than 50 local NMRAs are working independently across Africa to register medicines with different administrative and technical requirements, different registration processes, and limited transparency during the process [68]. Efforts toward regulatory harmonization are underway in Africa under the auspices of the African Medicines Regulatory Harmonization Programme—a consortium of partners including the WHO, Pan-African Parliament, New Partnership for Africa’s Development, the UK Department for International Development, the Clinton Foundation, and the Bill and Melinda Gates Foundation. Major stakeholders are NMRA representatives, the European & Developing Countries Clinical Trials Partnership, *the Deutsche Gesellschaft für Internationale Zusammenarbeit*, the European Medicines Agency, the International Federation of Pharmaceutical Manufacturers & Associations, and the African Regulatory Network. The African Medicines Regulatory Harmonization initiative aims to establish five to six regional groups (each with harmonized technical requirements) that will coordinate registration processes across the African continent. The regional groups will have standardized processes and documentation, as well as streamlined, faster, and more-reliable processes that are better aligned with the public health needs of each regional group. Progress has been made, especially in East Africa, with the WHO/East African Community (EAC) Medicines Regulatory Harmonization project (Burundi, Kenya, Tanzania, Uganda, Rwanda, and Zanzibar): four joint assessment sessions in 9 months have resulted in approvals in EAC NMRAs less than 2 months after joint acceptances by the EAC/WHO. Actions on regulatory harmonization are also underway in Southeast Asia via the Pharmaceutical Product Working Group of the Association of Southeast Asian Nations Consultative Committee for Standards and Quality and in the Asia–Pacific Economic Co-operation region via the Asia Pacific Harmonization Center [69, 70].

**Table 3:** How can the medicine registration processes in Africa be improved?

Today’s current environment	A harmonized future environment
~50 different NMRAs (working independently) to register medicines across Africa	~5 or 6 regional groups (each with harmonized technical requirements) coordinating registration across the entire African continent
Different administrative and technical requirements, processes, and procedures for medicines registration across NMRAs	Common (harmonized) registration documentation (format and technical requirements), procedures, and decision-making processes across African regional groups
No clear indication of the time taken, or the maximum times allowed, for regulators to assess and register medicines	Streamlined processes that are faster, more predictable, and better aligned to public health needs (in terms of prioritization, conditional approvals, etc.)
Limited transparency before or during the registration process	Transparent and clear procedures and a good understanding of registration requirements and processes by all stakeholders

Source: The New Partnership for Africa’s Development (NEPAD) and the World Health Organization (WHO). African Medicines Registration Harmonisation Initiative: Summary, Status and Future Plans [71]

Enforcement of drug regulations is another area in which collaboration is essential. National-level action against deliberately falsified medicines requires NMRAs to conduct collaborative investigations with police and customs. Operation Storm I and II, conducted in 2008 and 2009 in Cambodia, Indonesia, Laos, Myanmar, Singapore, Thailand, and Vietnam, offered an example of this sort of multisector collaboration. Coordinated by WHO and INTERPOL, these operations involved a synergistic partnership between customs authorities, police, NMRAs, and laboratories in participating countries [26]. Encouraging data from Cambodia suggest that measures to strengthen drug regulation and enforcement capacity and improve education and communication have reduced the circulation of falsified anti-malarial products, although substandard medicines remain common and are a specific threat for resistance [30].

The Global Fund has established a Joint Interagency Task Force (JIATF) to pro-actively engage with NMRAs and law enforcement authorities, and to provide information on the circulation of falsified medicines (based on its own data gathering and analysis), training, and analytic technologies [72]. Supporting NMRAs has also been defined as a priority for attention by the Global Steering Committee for the quality assurance of health products [72].

## Legal aspects

Action is needed at both the international and national levels to strengthen laws and law enforcement with regard to both falsified and substandard medicines. There have been calls for an international treaty, founded on considerations of public health (rather than intellectual property), to define and differentiate in law the different forms of poor-quality medicines, provide a framework for criminal prosecution (commensurate with the relative seriousness of the offences and intentions of the perpetrator), and harmonize regulatory standards [3, 18]. The 2013 protocol to eliminate the illicit trade in tobacco products within the Framework Convention on Tobacco Control [73] has been cited as a suitable model for this treaty.

At the national level, Attaran [35, 74] recently proposed a Model Law on Medicine Crime as a flexible template for any country to use to strengthen relevant laws on poor-quality medicines. This Model Law is consistent with the principles above and also has provisions concerning internet ‘pharmacies’, ‘whistle-blowers’, and unregistered medicines.

## Access, finance, and political commitment

In 2013, the United Nations urged all states to establish national health and regulatory infrastructures and domestic management capacities to ensure that all citizens have access to medicines that are affordable, safe, efficacious, and of good-quality, and for the international community to continue to assist in achieving this goal [75]. The WHO’s current global malaria strategy emphasizes the importance of providing universal access to quality-assured and appropriate anti-malarial medicines (together with diagnostics and vector control measures) as the first priority for countries with high or moderate malaria transmission rates [42]. This strategy needs to be extended into areas of low- and unstable transmission given that individuals in these settings have little acquired immunity and are more likely to suffer from severe malarial disease; furthermore, these environments are probable sources of drug-resistant parasites [76, 77]. Provision of good-quality medicines via public and private sector health services is important to prevent patients from turning to unreliable private sources [20]. Political commitment and financing is, therefore, vital to ensure that good-quality medicines are available and affordable [42].

## Call to action

Access to good-quality medicines is an essential human right and a top priority in the global fight against many life-threatening diseases including malaria [42]. The continued circulation of poor-quality (falsified and substandard) medicines in endemic regions is a key threat to future progress and public health as a whole. Although many actions are underway, important unmet needs remain and these warrant urgent attention and concerted multisector action, led and coordinated by a globally mandated organization at the international and national levels.

Given the current situation, urgent action is required by the international community across eight key areas:

1. Confer sustained *international* and *national* political commitment and financing to drive these and other measures necessary to ensure access to quality medicines and thereby protect public health.
2. Form a more comprehensive *international*, public health-orientated legal framework or treaty on poor-quality medicines, with statutes integrated into *national* laws.
3. Provide further *international* efforts to strengthen governance of global medicines supply chains, harmonize regulatory standards and related procedures, and facilitate international cooperation between NMRAs and law-enforcement bodies.
4. Develop harmonization of registration processes in *regional* centres of excellence with common registration documentation, procedures, and decision-making procedures that are better aligned with public health needs.
5. *Nationally*, develop NMRAs that are adequately prioritized, resourced, structured, and trained to allow them to perform all regulatory functions (including systematic surveillance, manufacturing oversight, quality assurance, registration, and enforcement) in collaboration with the other national partner authorities.
6. Invest in innovative technologies *globally* and *nationally* to support anti-malarial initiatives, particularly the detection of poor-quality medicines at all levels of the supply chain, and track-and-trace technologies that ensure a valid product from the manufacturing facility to the consumer.
7. Arrange specific *global* and *regional* support to increase the number of prequalified standardized reference laboratories that can detect poor-quality anti-malarial medicines and also drive the validation and scaling-up of new field technologies for surveillance and analysis, particularly in malaria-endemic countries via appropriate training and resourcing.
8. *National* and *regional* surveillance to be linked to effective routine and transparent reporting of incidents followed by an immediate withdrawal of products in the case of major findings by national authorities. Reporting of events should be made compulsory and a data-sharing policy involving public and private sectors, patient representatives, academics, non-government organizations, and international bodies should be developed accordingly.

While it is not the intention of this publication to allocate responsibilities to defined organizations and initiatives, questions on strategic roles must be discussed and clearly assigned for future coherent actions. These eight key areas are considered as the priorities for all stakeholders, including the scientific and medical community and health policymakers, and the joint actions of these groups will be critical in driving forward effective malaria elimination programmes and global health development.

## Conclusions

The continued circulation of poor-quality anti-malarial medicines poses a significant threat to global advances in combating malaria. There are many ongoing initiatives designed to address different aspects of the problem; however, it is imperative that key unmet needs are addressed with urgent multisector action. The role of the international community is vital in extending and strengthening actions across eight crucial areas and reinforcing the fight against malaria.

## Abbreviations

ACT: artemisinin-based combination therapy
API: active pharmaceutical ingredient
EAC: East African Community
INTERPOL: International Criminal Police Organization
JIATF: Joint Interagency Task Force
NMRA: National Medicine Regulation Authority
PQM: promoting the quality of medicines in developing countries
SSFFC: substandard, spurious, falsely labelled, falsified and counterfeit
USAID: United States Agency for International Development
USP: United States Pharmacopeial Convention
WHO: World Health Organization
WWARN: Worldwide Antimalarial Resistance Network
